# Proximal femoral nail antirotation versus cemented calcar-replacement hemiarthroplasty for unstable intertrochanteric fracture in elderly: an overall survival study

**DOI:** 10.3906/sag-2105-28

**Published:** 2021-12-16

**Authors:** Fatih DURGUT, Erdem ŞAHİN, Sadettin ÇİFTCİ, Bahattin Kerem AYDIN

**Affiliations:** 1 Department of Orthopaedics and Traumatology, Medical Faculty, Dicle University, Diyarbakir, Turkey; 2 Department of Orthopaedics and Traumatology, Erzurum Regional Training and Research Hospital, Erzurum, Turkey; 3 Department of Orthopaedics and Traumatology, Medical Faculty, Selçuk University, Konya, Turkey

**Keywords:** Unstable femur intertrochanteric femur fracture, cemented calcar-replacement hemiarthroplasty, proximal femoral nail

## Abstract

**Background/aim:**

The aim of this study is to compare the perioperative complications and overall survival of patients who underwent proximal femoral nail antirotation (PFNA) and patients who underwent cemented calcar-replacement hemiarthroplasty (CCRH) for unstable intertrochanteric fracture in patients aged 75 years and older.

**Materials and methods:**

A total of 94 patients who underwent PFNA or CCRH between 2010 and 2012 because of femur fracture (A2.2 and A2.3 according to the Arbeitsgemeinschaft für Osteosynthesefragen/Orthopaedic Trauma Association (AO/OTA) classification) were analyzed retrospectively. Hospitalization times, blood transfusion needs, reoperation rates, and overall survival were compared.

**Results:**

Forty-eight patients in the PFNA group and 46 patients in the CCRH group were included for analysis. There was no statistically significant difference between the two groups in terms of hospitalization times, blood transfusion needs, reoperation rates, and survival rates.

**Conclusions:**

Both PFNA and CCRH techniques can be used for surgical treatment of unstable intertrochanteric femur fractures.

## 1. Introduction

The increase in the average life expectancy in the world has led to an increase in the incidence of hip fractures. As the age progresses, the decrease in vision, muscle strength, and balance facilitates decrease and ultimately leads to hip fracture. In a 2050 perspective, it is estimated that the number of hip fractures can reach over 6 million [1] The intertrochanteric femoral fractures account for 45%–50% of hip fractures [2] and 50%–60% of these are of unstable fractures [3].

Surgical management of unstable intertrochanteric fracture remains challenging all over the world. At the present time, intramedullary (nails) and extramedullary (screw or plates) fixations and total or partial arthroplasty are used in the treatment. Especially patients over 75 years of age have poor bone quality due to osteoporosis, so complications are more common such as nonunion, femoral head collapse, and metal failure.[4] Most studies in the literature recommend proximal femoral nail and hemiarthroplasty as the first surgical choices for the surgical treatment of unstable intertrochanteric fracture [5,6].

The objective of this study is to compare the perioperative complications and overall survival of patients who underwent proximal femoral nail antirotation (PFNA) and patients who underwent cemented calcar-replacement hemiarthroplasty (CCRH) for unstable intertrochanteric fracture in patients aged 75 years and older.

## 2. Materials and methods

Institutional review board approval was obtained for this cohort study. The patients who underwent PFNA or CCRH between 2010 and 2012 because of intertrochanteric femur fracture (A2.2 ve A2.3 according to the Arbeitsgemeinschaft für Osteosynthesefragen/Orthopaedic Trauma Association (AO/OTA) classification) were analyzed retrospectively. The patients 75 years and more were included in the study. Exclusion criteria were age <75 years, pathologic fractures, bilateral fractures, treatment with a method other than PFNA or CCRH, metabolic bone disease, multiple trauma.

Ninety-four patients who received surgical treatment for unstable femoral intertrochanteric fractures were included in the study. Forty-six patients underwent CCRH and forty-eight patients underwent PFNA. All operations were performed by the same surgical team. Hospitalization times, blood transfusion needs, reoperation rates, and overall survival were compared.

The patients were followed up at 6 weeks, 3 months and 6 months for clinical and radiological examinations. Complications diagnosed during the controls were noted. Also patients or their relatives were called and if the patients died, the dates of death were noted.

All statistical analyses were performed using SPSS version 15.0 software (SPSS Inc., Chicago, IL) and p < 0.05 level was considered as significant. Conformity of the data to normal distribution was evaluated with the Kolmogorov-Smirnov test. The Mann Whitney U test was used for two-group comparisons of nonnormally distributed parameters. Categorical variables were analysed using the chi-square test. Kaplan Meier method for survival analysis was used.

## 3. Results

Ninety-four patients with unstable intertrochanteric femur fractures included in the study were evaluated retrospectively. All patients were 75 years of age or more. The PFNA group included 28 male (58.3%) and 20 female (41.7%) patients with a median age of 80 years (range, 75–90). The CCRH group included 14 male (30.4%) and 32 female (69.6%) patients with a median age of 83 years (range, 75–89). The demographical data were normally distributed and there was no significant difference between the study cohorts with regards to age and gender (Table 1).

The average time from hospitalization to the operation was 2.5 days (median) (range, 1–6) in the PFNA group and 3 days (median) (range,1–6) in the CCRH group. The median time from operation to discharge was 2.3 days (range, 1–5) in the PFNA group and 3 days (range, 2–7) in the CCRH group. There was no statistically significant difference between the two groups in the average length of preoperative hospital stay. Postoperative hospital stay was also found to be significantly less in the PFN group (Table 2).

While 32 (66.7%) of the patients who underwent PFNA did not need a postoperative blood transfusion, 4 patients (8.3%) transferred 1 unit and 12 patients (25%) transferred 2 units of blood transfusion. While 20 (43.5%) patients who underwent CCRH did not need a postoperative blood transfusion, 4 patients (8.7%) transferred 1 unit, 12 patients (26.1%) transferred 2 units and 10 (21.7) patients transferred 3 units of blood transfusion. No statistically significant difference was determined between the groups in respect of the blood transfusion requirement.

Revision surgery was performed in 2 patients (8.3%) in the PFNA group (femoral head collapse) and in 8 patients (20%) in the CCRH group (four dislocations, two periprosthetic fractures, two deep infections). There was no statistically significant difference between the two groups in the revision surgery (Table 2).

Sixteen of the patients who underwent CCRH were alive and 30 patients died. For these patients, the 1-year survival rate was 69.6%, the 2-year survival rate was 69.6%, the 3-year survival rate was 60.9%, the 5-year survival rate was 39.1% and the 10-year survival rate 34.8%. Median survival is 119 months (112–124) (Figure). Twenty of the patients who underwent PFNA were alive and 28 patients died. For these patients, the 1-year survival rate was 95.8%, the 2-year survival rate was 65%, the 3-year survival rate was 70.8%, the 5-year survival rate was 70.8%, and the 10-year survival rate was 45.1%. Median survival is 114 months (91.4–128.8) (Figure). There was no statistically significant difference between survival times (p = 0.26).

## 4. Discussion

The incidence of intertrochanteric fractures in the elderly continues to increase recently. Osteoporosis is a common problem in patients 75 years of age or more that decreases bone quality. Additional comorbidities are also available in these patients; all these increase the complications that may occur after surgery in patients with hip fractures. The 1-year mortality for hip fractures ranges from 14% to 36% [7] and so preoperative fracture must be carefully evaluated and a personalized protocol must be established for each patient in order to develop an appropriate treatment plan. The purpose of the treatment of hip fractures in the elderly is to restore the preoperative ambulatory status with the lowest possible surgical and medical complication rate.

In the elderly population, intertrochanteric femoral fractures account for 45%–50% of all hip fractures [2] and 50%-60% of these are of unstable fractures [3]. Especially unstable intertrochanteric fractures are characterized by severe comminution and displacement and so anatomic reduction of the fractures is difficult to achieve and maintain.

Numerous implant models continue to be developed for intertrochanteric fracture surgery. In the stable intertrochanteric femur fractures, sliding nail maintains priority[8]. Most authors recommend that PFNA has good biomechanical results due to its antirotation, anticompression, and antitension abilities, so it can be used as a suitable treatment modality in unstable intertrochanteric femoral fractures [9].

Recently, primary arthroplasty with a low failure rate has been used as an effective treatment for unstable fractures [10]. Primary arthroplasty increases the activity level of patients by providing early weight-bearing and prevents the development of potentially fatal complications [11].

Since fracture reduction is extremely difficult, multiple fluoroscopy images are required to evaluate the fracture during surgery in patients with proximal femoral nails. However, in cases with arthroplasty, a few control fluoroscopy images are sufficient.

In our study, we obtained similar results with the literature. As revealed in a recent meta-analysis, the postoperative hospital stay was shorter in the PFNA group in our study [12]. There is no statistically significant difference between the other parameters we compare. The lack of statistical difference between survival times may be due to the low number of patients included in the study. Therefore, studies with larger series may be more meaningful.

Although we could not find a statistically significant difference in our study, the surgeon spends more time on bleeding control in arthroplasty cases compared to proximal femoral nail cases.

The recent development of cement technology has reduced the incidence of a pulmonary embolism that can develop during cement placement [13,14]. However, this is still known to pose a risk in cemented cases.

In the follow-up of the patients included in our study, we observed that the patients in the CCRH group has faster mobilization in the early postoperative period. On the 1st day after the operation, the patients were able to walk with the help of a walker in the CCRH group. Being able to press on the affected extremity early can be considered as an advantage. However, functional capacities in the postoperative 6th month were similar in both groups.

Limitations of the study include not comparing the operation time and not using an objective parameter such as the Harris hip score while evaluating hip functions. In addition, the small number of patients in both groups and the short follow-up period can be added to these limitations. Not knowing the homogeneity of the groups in terms of comorbidities that may contribute to mortality in patients or ASA scores constitutes an important limitation.

In conclusion, our study shows that both PFNA and cemented calcar-replacement hemiarthroplasty are safe and have similar results for the treatment of intertrochanteric femur fractures. Similar studies or meta-analyses to be conducted will give an idea for the correct treatment option for patients of 75 years of age and more with unstable intertrochanteric fractures. However, since each patient has different comorbidities, it would be appropriate to develop a personal treatment protocol.

## Figures and Tables

**Figure:**
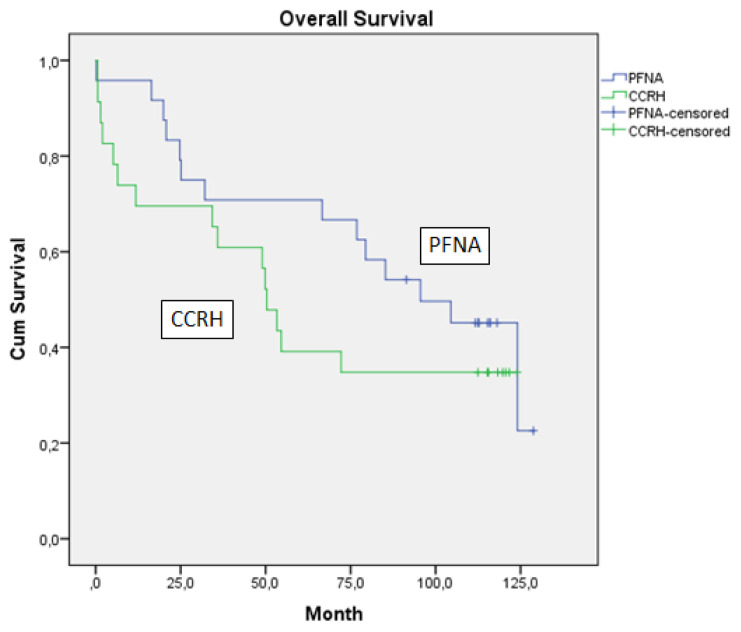
Overall survival.

**Table 1:** Demographics of the patients.

	PFNA	CCRH	p value
**Age (median, range)**	80 (75–90)	83 (75–89)	0.06
**Gender (N)**			
Male	28	14	0.05
Female	20	32	

PFN, proximal femoral nail antirotation group; CCRH, cemented calcar-replacement hemiartroplasty group

**Table 2:** Postoperative results.

	PFN	CCRH	p value
**Preoperative hospitalization (mean day)**	2.5	3	0.32
**Postoperative hospitalization (mean day)**	2.3	3	0.01
**Revision surgery (N)**	1	4	0.14

PFNA, proximal femoral nail antirotation group; CCRH, cemented calcar-replacement hemiartroplasty group
